# Supplementary material for: Programmable conjugative CRISPR interference targeting genotoxin in the gut
Source: bioRxiv. 2025 Oct 13:2025.10.13.682122. Preprint. [Version 1] doi: 10.1101/2025.10.13.682122 (PMC12632936; doi:10.1101/2025.10.13.682122)
Supplement: Supplement 1 [file media-1.pdf]

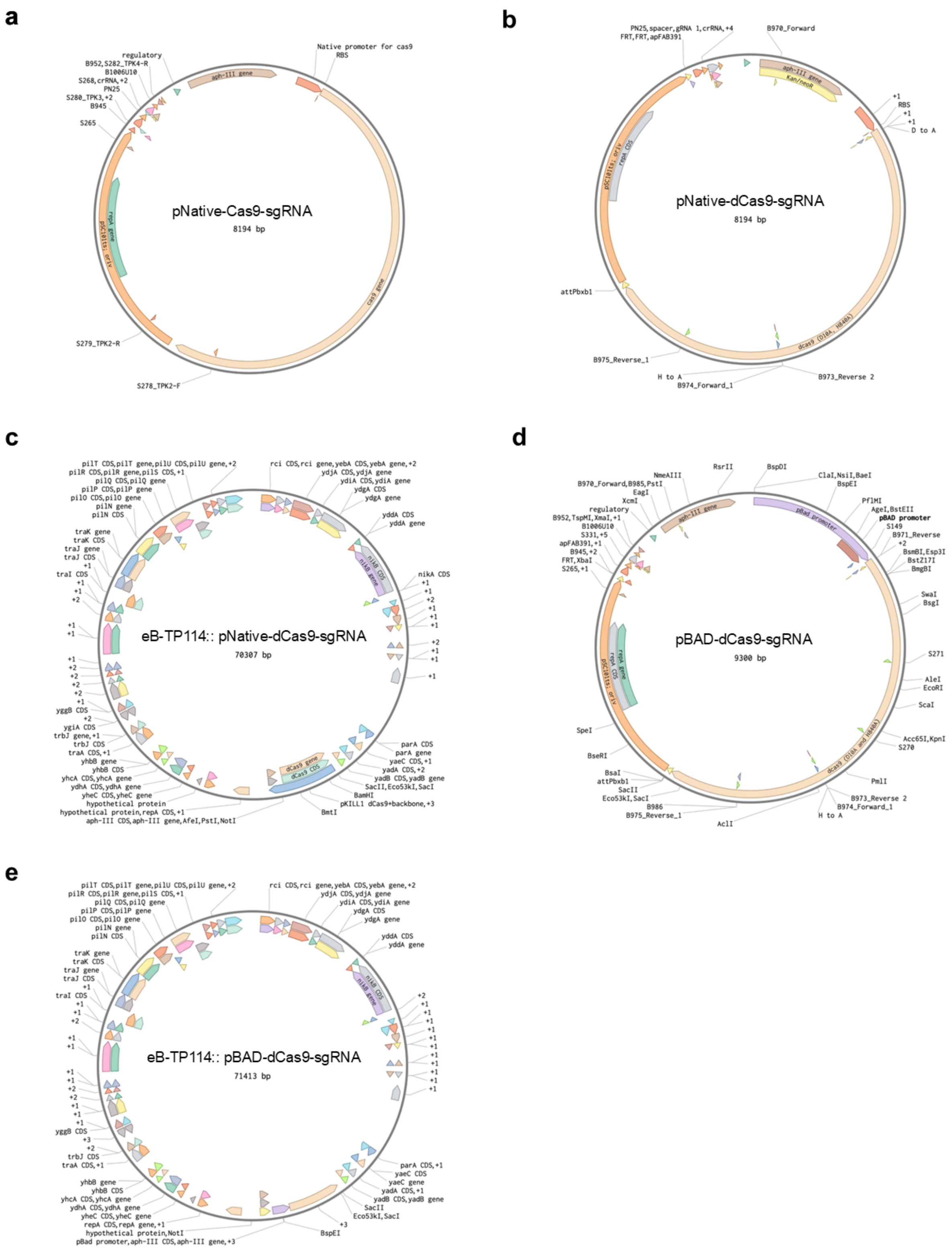

Extended Figure 1, Maps of replicative and conjugative plasmids.

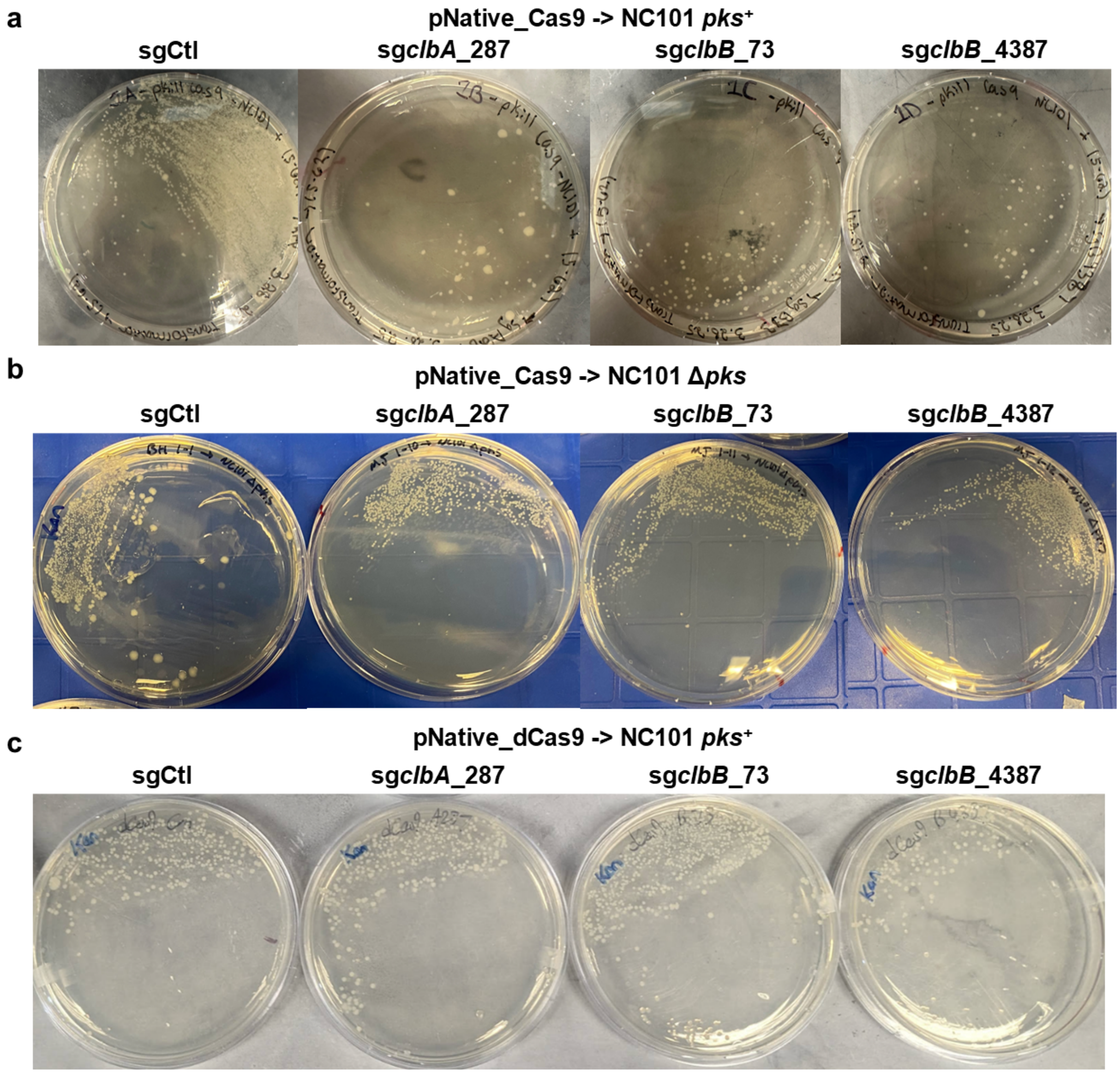

Extended Figure 2, Colony growth on lysogeny broth (LB) agar plates with kanamycin antibiotics shows the antimicrobial effects of pNative_Cas9 plasmids with *pks*-targeting sgRNA (**a**). This antimicrobial effect is *pks*-dependent, as transformation of NC101 Δ*pks E. coli* with pNative_Cas9 plasmids shows no effect on colony growth on LB agar plates with kanamycin (**b**). pNative_dCas9 shows no such antimicrobial effects (**c**). Results are presentative of three independent replicates.

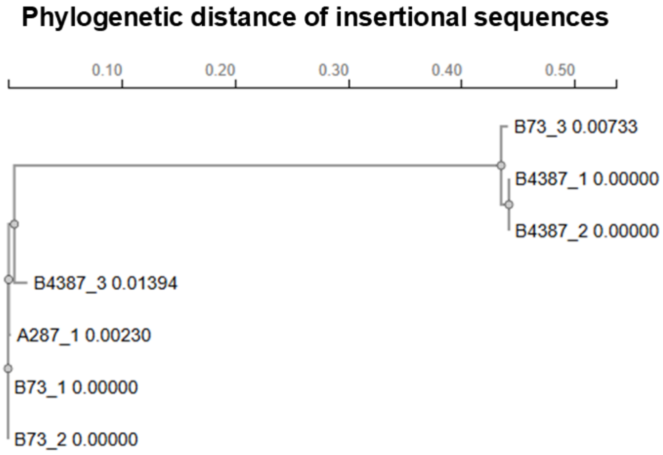

Extended Figure 3, Phylogenetic distance after alignment of insertional sequences discovered within the open reading frame of the Cas9 gene. The sequence alignment and phylogenetic tree were generated by Clustal Omega (multiple sequence alignment).

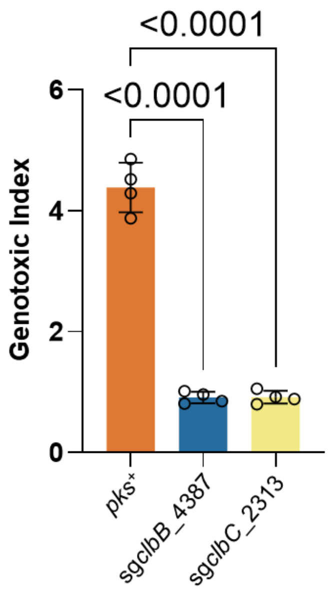

Extended Figure 4, NC101 *pks*$^{+}$ cells conjugated with eB-TP114-pBAD_dCas9 constructs cultured in anaerobic conditions prior to the HeLa co-culture can effectively lower the genotoxic index as compared to positive controls. MOI = 100, n = four technical repeats per independent biological replicate, N = two independent biological replicates. Data are means ± SD. Significance values calculated using one-way ANOVA with Dunnett's multiple comparisons test.

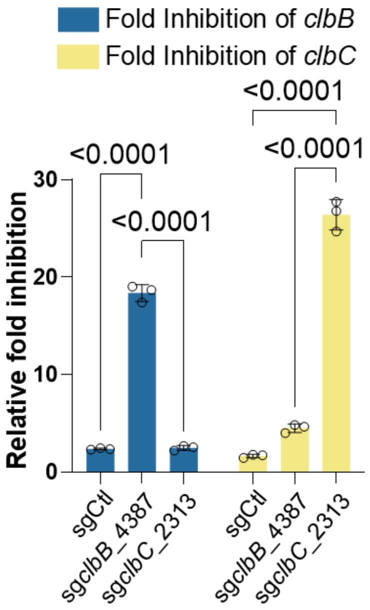

Extended Figure 5, $2^{-\Delta\Delta Ct}$ qPCR analysis of *pks*$^+$ *E. coli* Nissile conjugated with eB-TP114-pBAD_dCas9 plasmids show highly specific targeting of the *pks* island. n = three technical repeats per independent biological replicate, N = two independent biological replicates. Data are means ± SD. Significance values calculated using two-way ANOVA with Tukey's multiple comparison test.

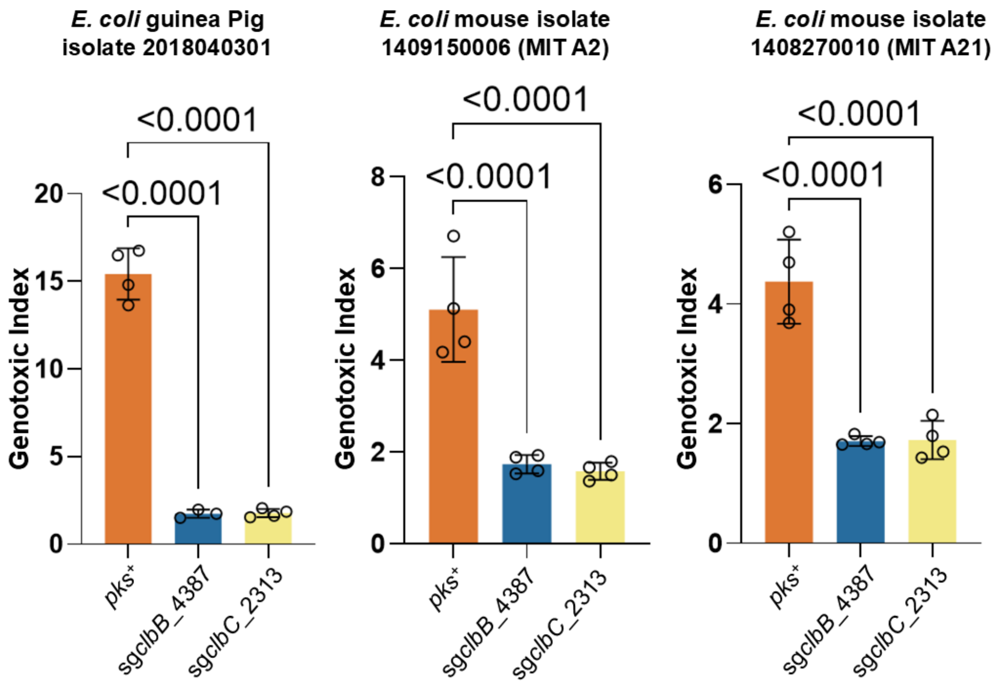

Extended Figure 6, eB-TP114-pBAD_dCas9 strains transformed into *pks*+ bacteria extracted from different mammalian hosts significantly lower genotoxicity in HeLa cells after 4 h co-culture as compared to positive controls. MOI = 100, n = four technical repeats per independent biological replicate, N = two independent biological replicates. Data are means ± SD. Significance values calculated using one-way ANOVA with Dunnett's multiple comparisons test.

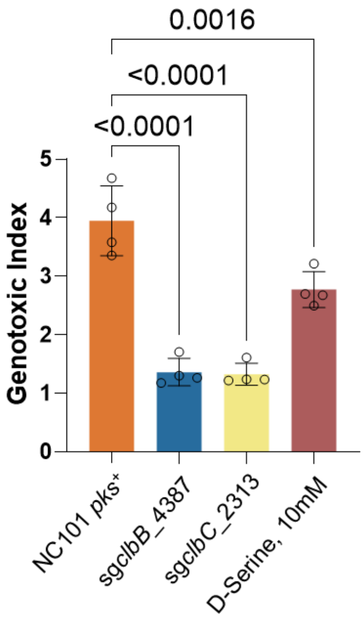

Extended Figure 7, NC101 *pks*$^{+}$ conjugated with eB-TP114-pBAD_dCas9 constructs can more effectively lower the genotoxic index as compared to positive controls and the small molecule inhibitor D-Serine. MOI = 100, n = four technical repeats per independent biological replicate, N = two independent biological replicates. Data are means ± SD. Significance values calculated using one-way ANOVA with Dunnett's multiple comparisons test.

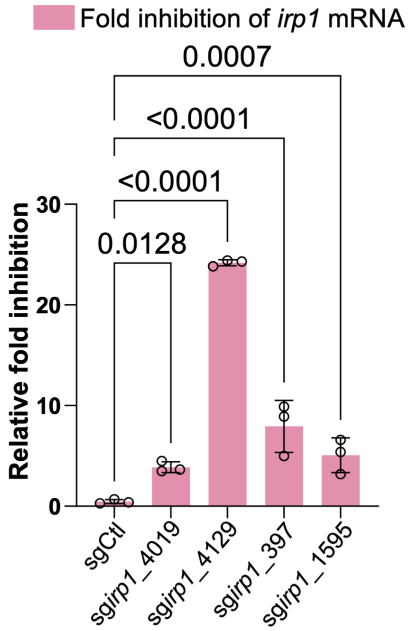

Extended Figure 8, *pks*$^{+}$ NC101 *E. coli* are conjugated with eB-TP114-pBAD_dCas9 plasmids encoding four different *irp1*-specific sgRNAs or the non-targeting sgCtl. $2^{-\Delta\Delta Ct}$ qPCR analysis of *irp1* mRNA shows specific targeting of the *irp1* gene, which is necessary for the biosynthesis of yersiniabactin. n = three technical repeats per independent biological replicate, N = two independent biological replicates. Data are means ± SD. Significance values calculated using two-way ANOVA with Dunnett's multiple comparison test.

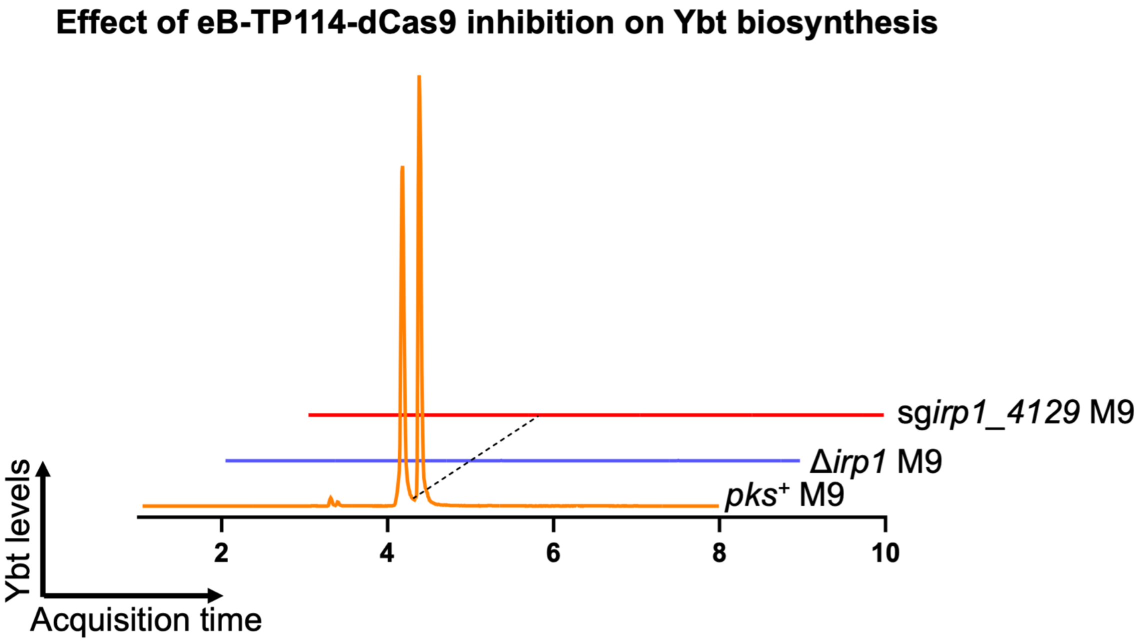

Extended Figure 9, Liquid chromatography-mass spectrometry-quadrupole time of flight (LC-MS-QTOF) analysis of *pks*$^{+}$ NC101 *E. coli* conjugated with eB-TP114-pBAD_dCas9-sg*irp1*_4129 show complete disruption of the biosynthesis of yersiniabactin (ybt) through the silencing of the *irp1* gene as compared to positive and negative controls grown in minimal M9 broth (M9). Results are representative of two independent experiments.

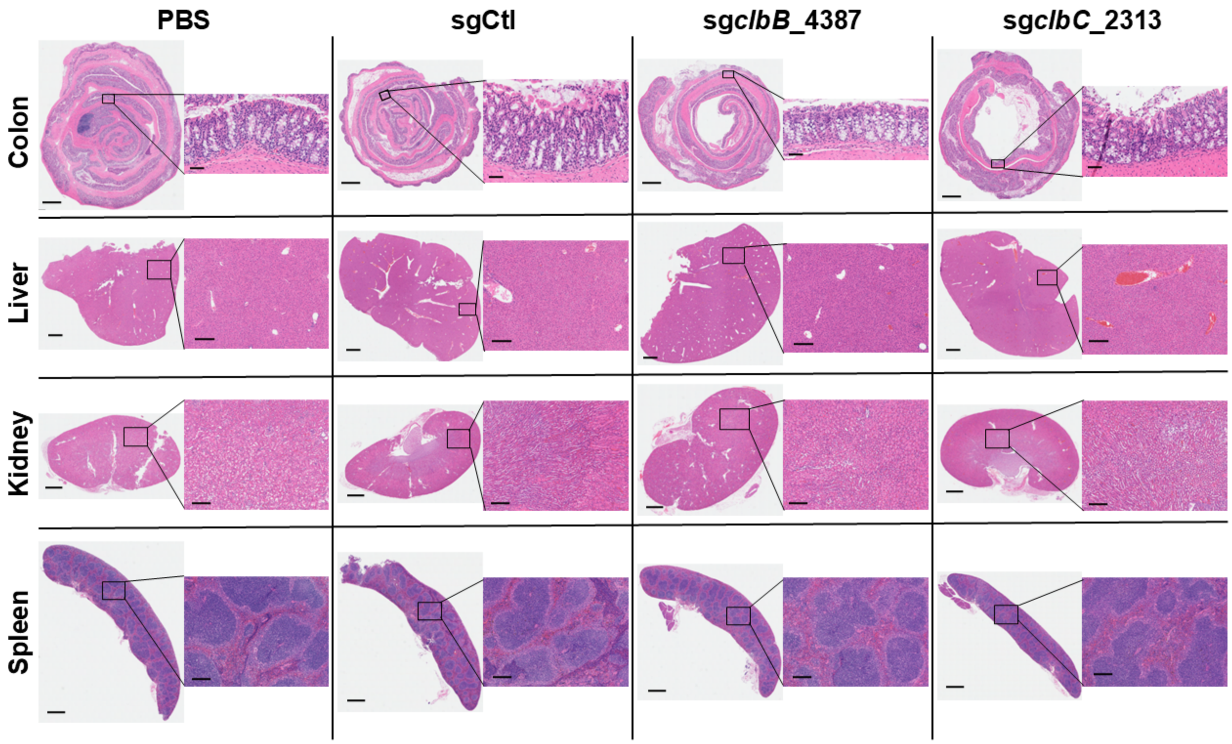

Extended Figure 10, H&E staining of colon Swiss rolls, livers, kidneys, and spleens show similar phenotypes with no obvious signs of inflammation. Images are representative samples from each treatment group. Scale bars on colon full zoom images are 800µm; scale bars on colon insets are 50µm. Scale bars on liver, kidney, and spleen full zoom images are 1000µm; scale bars on liver, kidney, and spleen insets are 200µm.

1070

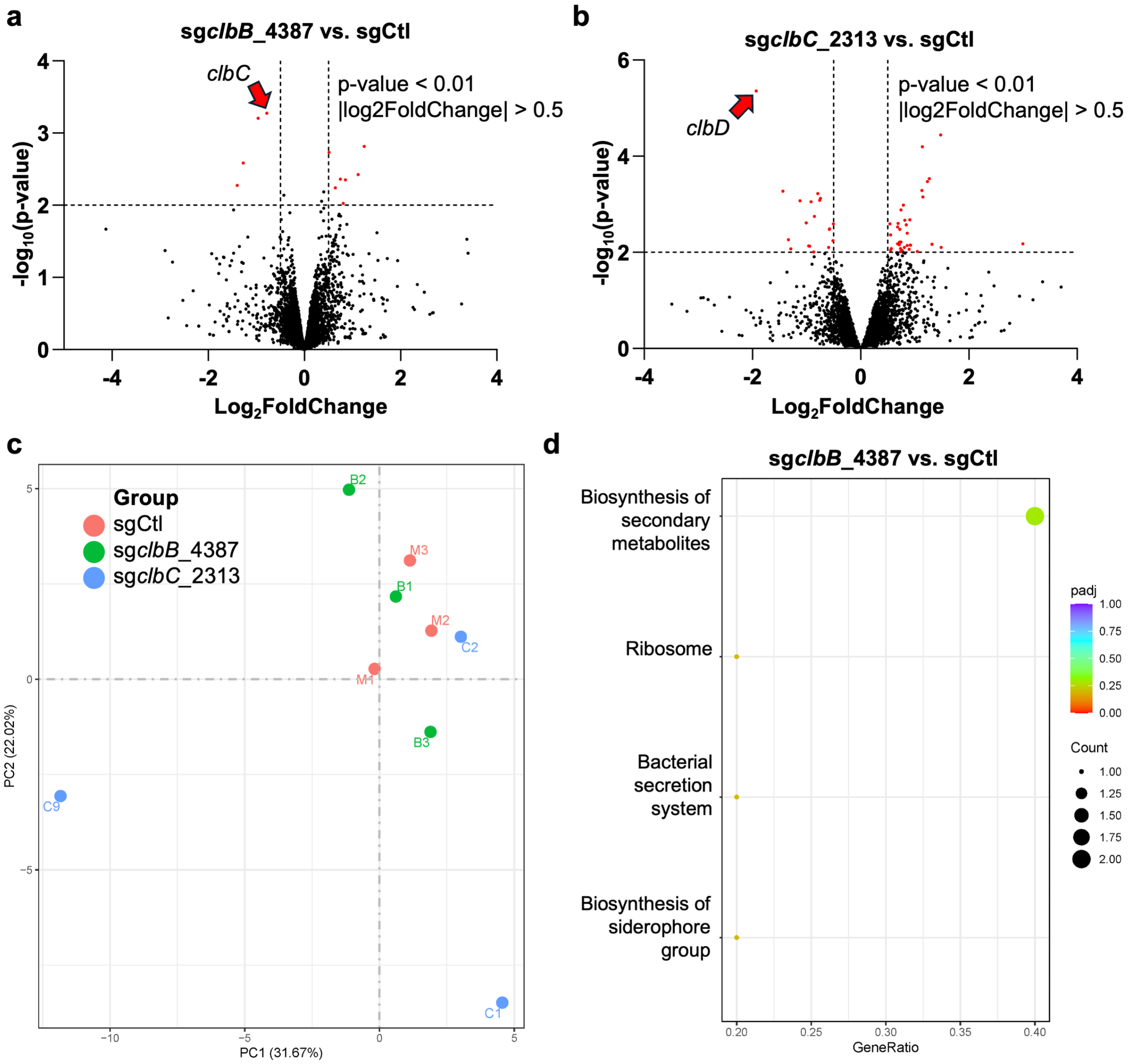

Extended Figure 11, RNAseq analysis of sg*clbB*_4387 and sg*clbC*_2313 vs. sgCtl show that *clbC* and *clbD* are the two genes most statistically significantly downregulated (**a** and **b**, respectively), but sg*clbC*_2313 significantly affects the expression of many more genes than sg*clbB*_4387 (19 downregulations and 35 upregulations vs. 4 downregulations and 7 upregulations, respectively). Principal component analysis reinforces this observation, as sgCtl and sg*clbB*_4387 are much more closely clustered than sg*clbC*_2313 (**c**). The associated KEGG plots show that sg*clbB*_4387 downregulates the biosynthesis of secondary metabolites (including, in this case, colibactin) (**d**).

1071

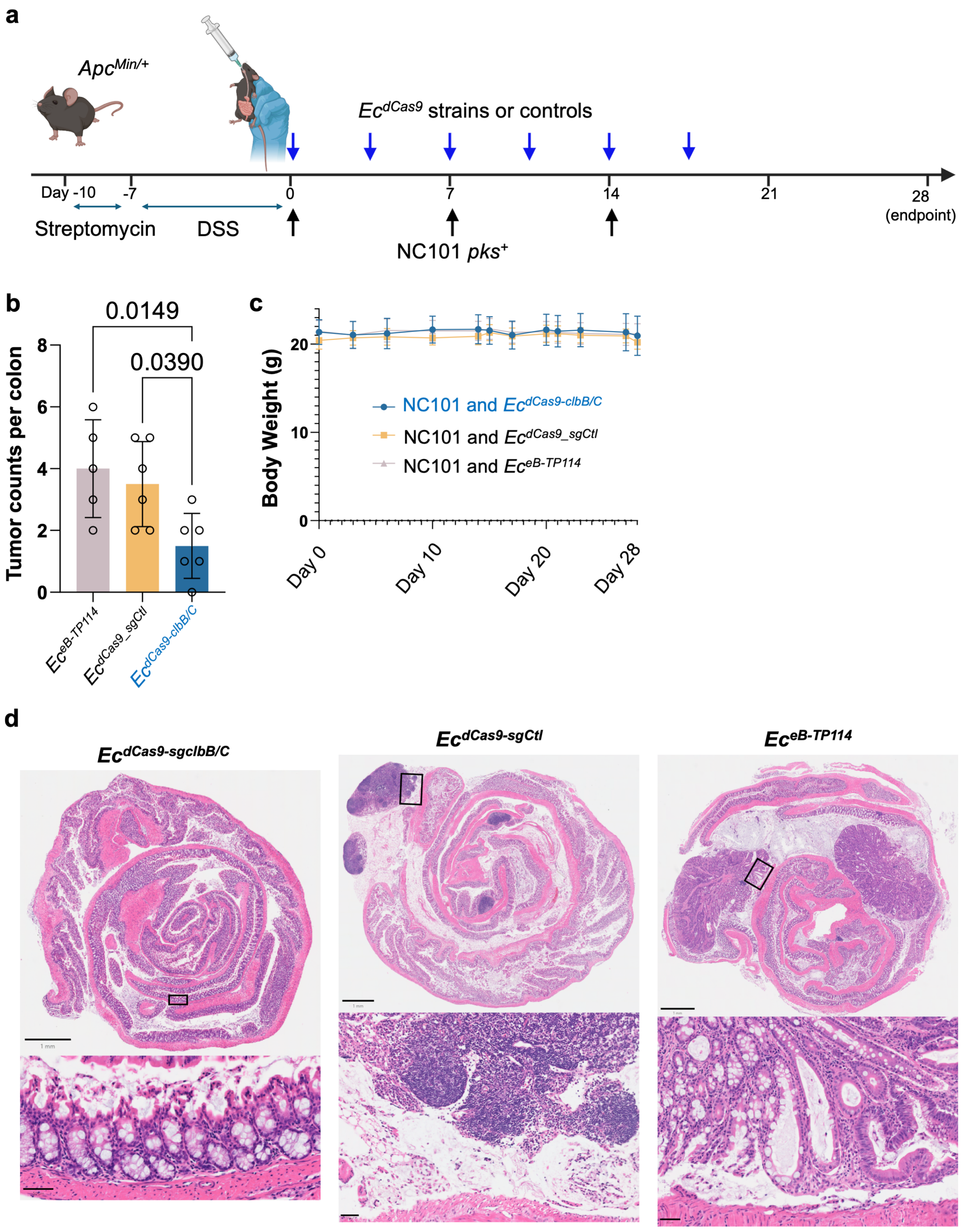

Extended Figure 12, a, the cocktail strategy combining equal mixture of *Ec*$^{dCas9\text{-}sgclbB\_4387}$ and *Ec*$^{dCas9\text{-}sgclbC\_2313}$ in DSS/*Apc*$^{Min/+}$ mice. Mice received one of three donor strains by oral gavage: (1) *Ec*$^{eB\text{-}TP114}$ (eB-TP114 lacking dCas9 and sgRNA), (2) *Ec*$^{dCas9\text{-}sgCtl}$, and 3) an equal mixture of *Ec*$^{dCas9\text{-}sgclbB\_4387}$ and *Ec*$^{dCas9\text{-}sgclbC\_2313}$, denoted as '*Ec*$^{dCas9\text{-}clbB/C}$'). **b**, *Ec*$^{dCas9\text{-}clbB/C}$ significantly

lowers tumor counts at time of sacrifice compared to $Ec^{eB\text{-}TP114}$ or $Ec^{dCas9\text{-}sgCtl}$. Data are means ± SD. Significance values calculated using one-way ANOVA with Dunnett's multiple comparisons test. **c**, Body weight changes over the course of treatment. **d**, representative H&E staining of colon Swiss rolls. Full image scale bars are 1mm, inset zoom bars are 50 µm.

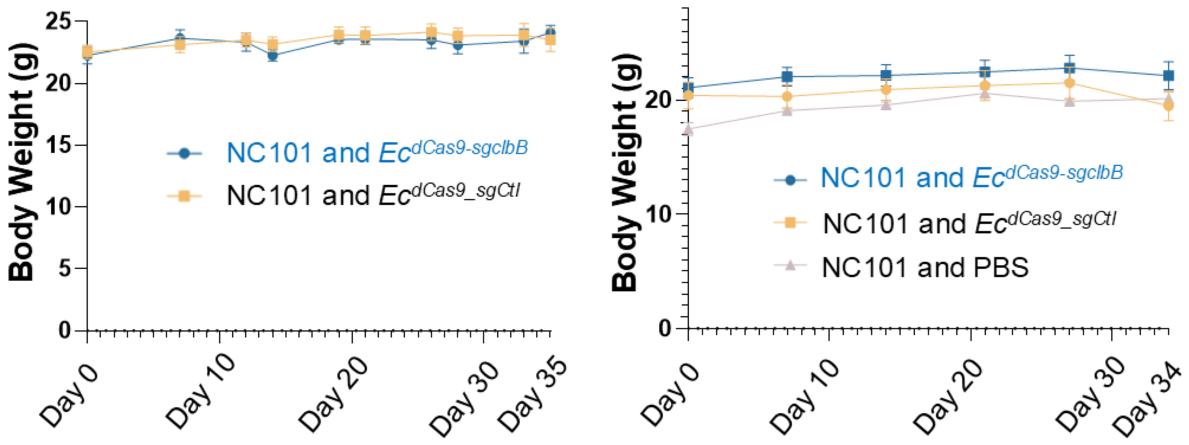

Extended Figure 13, Body weight records from the two *in vivo* efficacy studies comparing *Ec*$^{dCas9\text{-}sgclbB\_4387}$ and *Ec*$^{dCas9\text{-}sgCtl}$ show stable weights during the experimental timeframes.

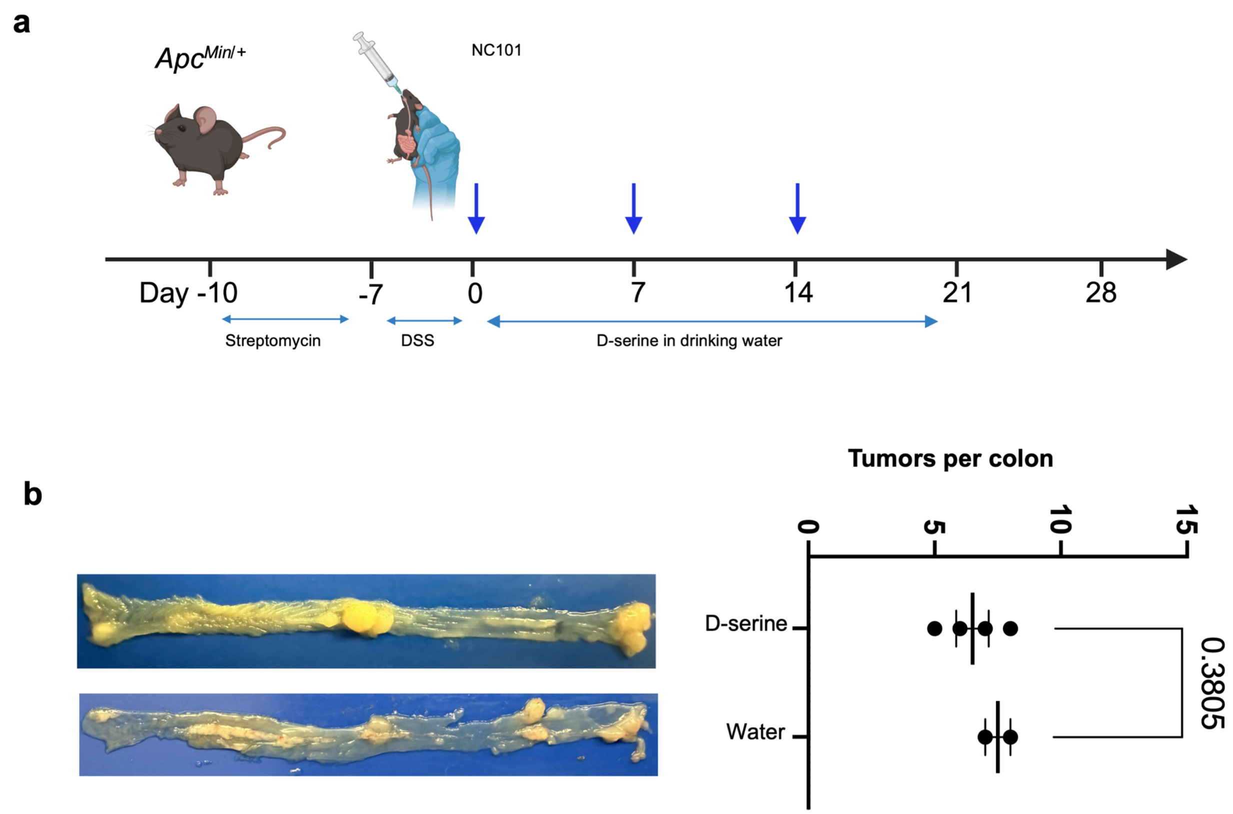

Extended Figure 14, D-serine supplementation does not suppress NC101-induced tumorigenesis compared with water controls. **a**, Timeline of DSS/*Apc*$^{\mathrm{Min/+}}$ mice treated with NC101 in combination with D-serine in drinking water. *Apc*$^{\mathrm{Min/+}}$ mice were pretreated with 2 g/L streptomycin in drinking water for three days for bacterial colonization, followed by 2% DSS in water for one week to induce colitis. Next, the mice were infected with NC101 weekly for three weeks, with concurrent 600 mg/L D-serine or water treatment. **b**, Representative tumors in the colon on day 28 and quantification of colonic tumor counts. Data are means ± SD using two-sided unpaired t test.

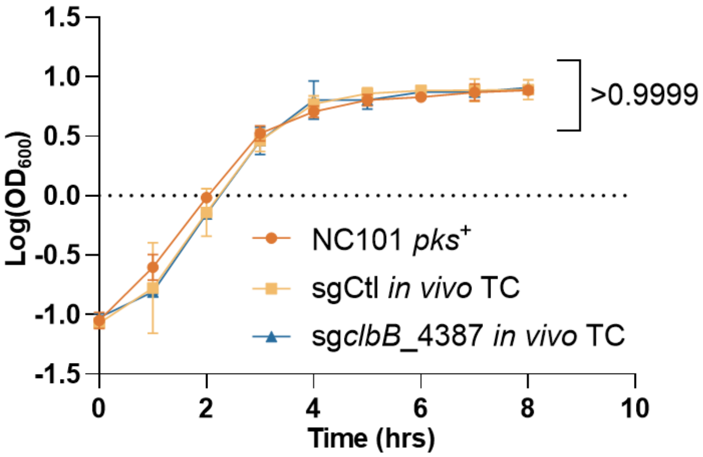

Extended Figure 15, Growth curve analysis of *pks*$^{+}$ NC101 *E. coli* and *in vivo* transconjugates (TC) show no statistically significant difference between cultures. n = 2 technical repeats, N = 2 biological replicates. Data are means ± SD. Significance values calculated using nonlinear regression analysis.

1078

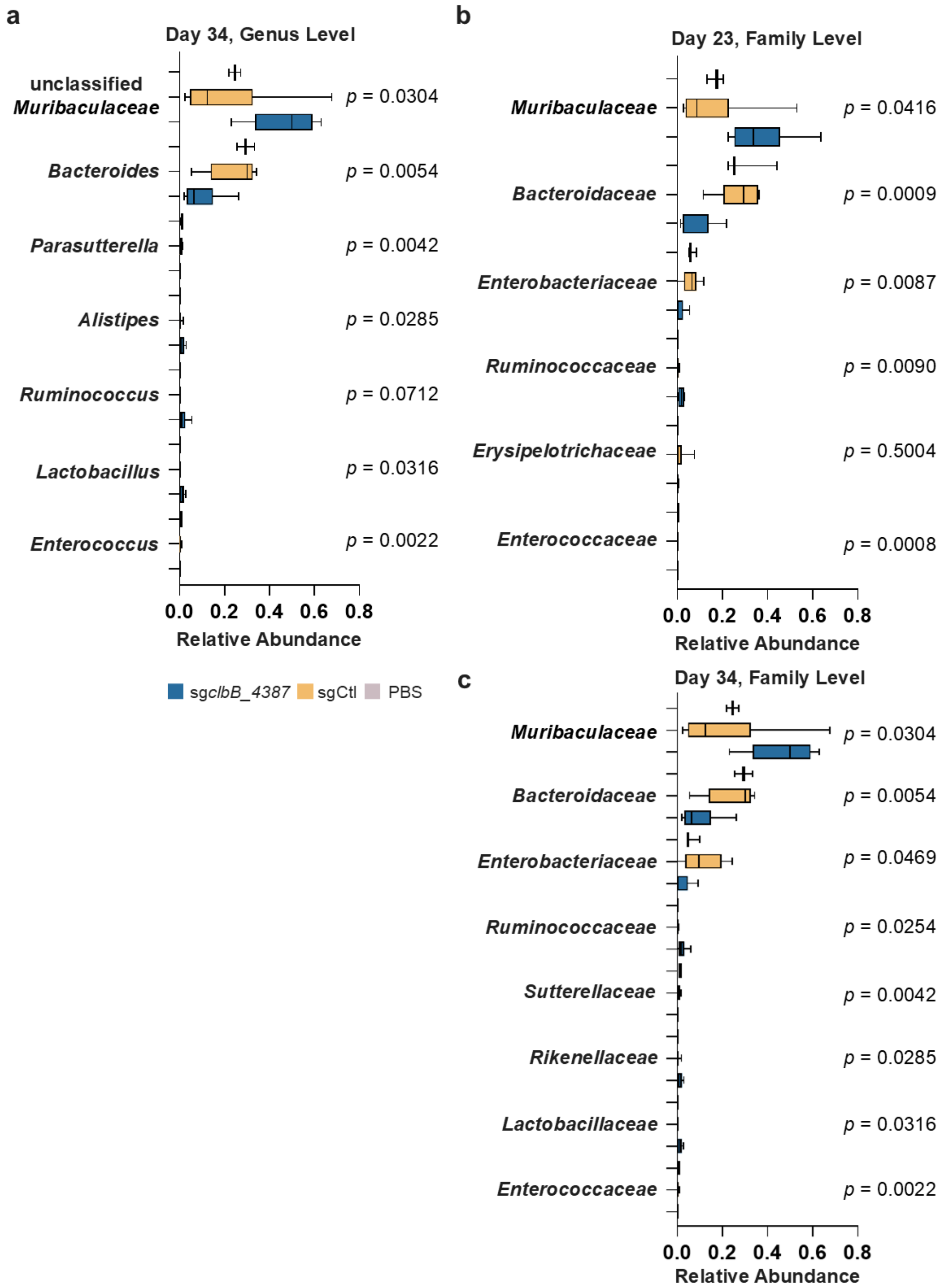

Extended Figure 16, Top differential abundance (%) at the genus level (**a**) and family level (**b** and **c**) from fecal microbiota in different groups at Day 23 (**b**) and Day 34 (**a** and **c**). The family level shows significant reduction of *Enterobacteriaceae* by $Ec^{dCas9\text{-}sgclbB\_4387}$ compared to the vehicle and $Ec^{dCas9\text{-}sgCtl}$ control at both mid and endpoints. In box plots, the bounds of the box

indicate the 25th–75th percentiles, the center line indicates the median, and whiskers extend from the minimum to the maximum values. Statistical values calculated using one-way ANOVA.

1084

a

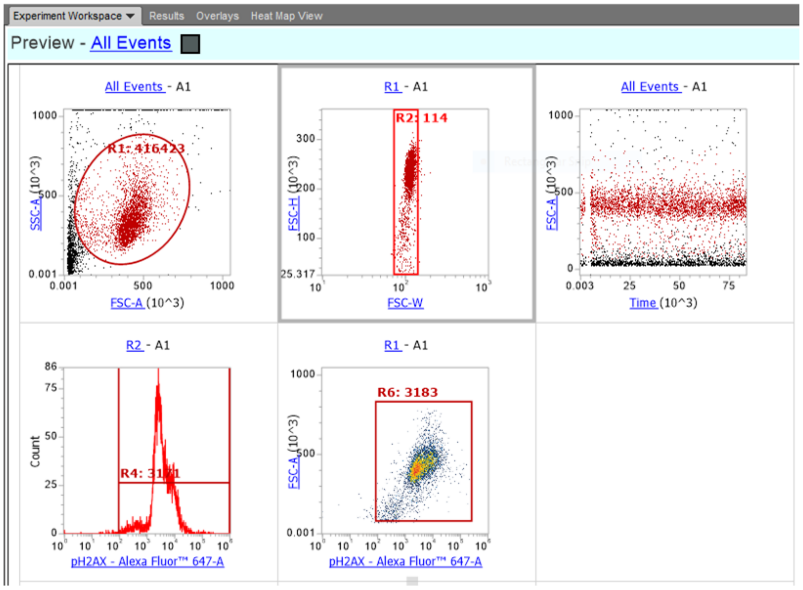

b

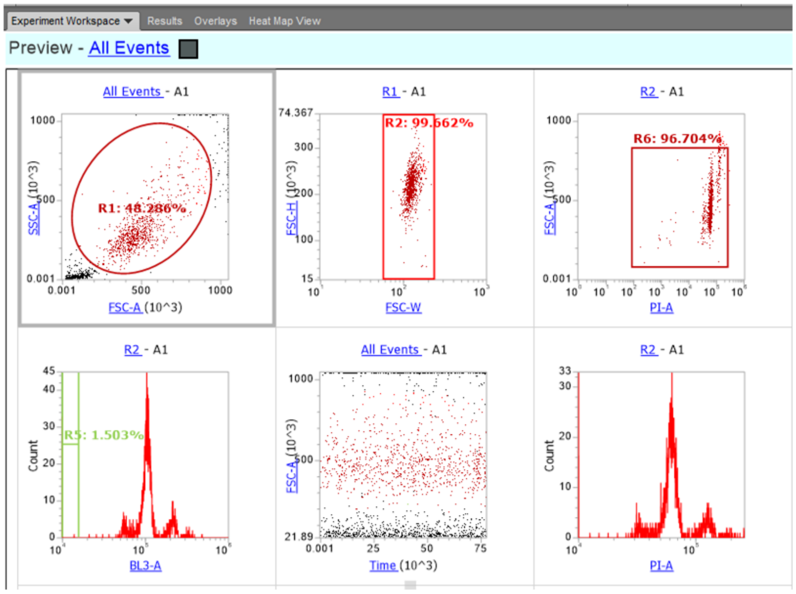

Extended Figure 17, Flow cytometry gating for γH2AX analysis (**a**) and cell cycle analysis (**b**).

Table S1, Alignment of insertional sequences discovered within the open reading frame of the Cas9 gene.

```
CLUSTAL O(1.2.4) multiple sequence alignment


B73_3        GGCAAAGCACTGAGAGATCCCCTCATAATTTCCCCAAAGCGTA---ACCATGTGT----G   53
B4387_1      TGCTAAGTCCTGAGAGATCCCCTCATAATTTCCCCAAAACGTA---ACCATGTGT----G   53
B4387_2      TGCTAAGTCCTGAGAGATCCCCTCATAATTTCCCCAAAACGTA---ACCATGTGT----G   53
A287_1       TACTCAATACTGATGAATCCCCTAATGATTTTTATCAAAATCATTAAGTTAAGGTAGATA   60
B73_1        TGCTAAGTCCTGATGAATCCCCTAATGATTTTTATCAAAATCATTAAGTTAAGGTAGATA   60
B73_2        TGCTAAGTCCTGATGAATCCCCTAATGATTTTTATCAAAATCATTAAGTTAAGGTAGATA   60
B4387_3      -CAAATAGCGCTACTAATCCCCTAATGATTTTGGTAAAAATCATTAAGTTAAGGTGGATA   59
                         *   ******* ** ****     **    *   *      **

B73_3        AATAAATTTTGAGCTAGT---AGGGTT-----------------GCAGCCACGAGTAAGT   93
B4387_1      AATAGATTTTGAGTAAGC---AGGGTT-----------------GCAGCCACGAGTGAGT   93
B4387_2      AATAGATTTTGAGTAAGC---AGGGTT-----------------GCAGCCACGAGTGAGT   93
A287_1       CACATCTTGTCATATGATCAAATGGTTTCGCCAAAAATCAATAATCAGACAACAAAATGT   120
B73_1        CACATCTTGTCATATGATCAAATGGTTTCGCCAAAAATCAATAATCAGACAACAAAATGT   120
B73_2        CACATCTTGTCATATGATCAAATGGTTTCGCCAAAAATCAATAATCAGACAACAAAATGT   120
B4387_3      CACATCTTGTCATATGATCAAATGGTTTCGCGAAAAATCAATAATCAGACAACAAGATGT   119
              * *  ** * *         * ****                  *** **  *    **

B73_3        CTTCCCTTGTTATTG------------TGTAGCCAGAATGCCGCAAAACTTCCATGCCTA   141
B4387_1      CTTCCCTTGTTATTG------------TGTAGCCAGAATGCCGCAAAACTTCCATGCCTA   141
B4387_2      CTTCCCTTGTTATTG------------TGTAGCCAGAATGCCGCAAAACTTCCATGCCTA   141
A287_1       GCGAACTCGATATTTTACACGACTCTCTTTACCAATTCTGCCCCGAATTA--C--ACTTA   176
B73_1        GCGAACTCGATATTTTACACGACTCTCTTTACCAATTCTGCCCCGAATTA--C--ACTTA   176
B73_2        GCGAACTCGATATTTTACACGACTCTCTTTACCAATTCTGCCCCGAATTA--C--ACTTA   176
B4387_3      GCGAACTCGATATTTTACACGACTCTCTTTACCAATTCTGCCCCGAATTA--C--ACTTA   175
                  ** * ****             * ** * *   **** * **     *   * **

B73_3        AGCGAACTGTTGAGAGTACGTTTCGATTTC-TGACTGTGTTAGCCTGGAAGTGCTTGTCC   200
B4387_1      AGCGAACTGTTGAGAGTACGTTTCGATTTC-TGACTGTGTTAGCCTGGAAGTGCTTGTCC   200
B4387_2      AGCGAACTGTTGAGAGTACGTTTCGATTTC-TGACTGTGTTAGCCTGGAAGTGCTTGTCC   200
A287_1       AAACGACTCAACAGCTTAACGTTGGCTTGCCACGCCTTACTTGACTGTAAAA----CTCT   232
B73_1        AAACGACTCAACAGCTTAACGTTGGCTTGCCACGCCTTACTTGACTGTAAAA----CTCT   232
B73_2        AAACGACTCAACAGCTTAACGTTGGCTTGCCACGCCTTACTTGACTGTAAAA----CTCT   232
B4387_3      AAACGACTCAACAGCTTAACGTTGGCTTGCCACGCATTACTTGACTGTAAAA----CTCT   231
             *    ***    **  **   ** * ** *    *  *  * * *** **       **

B73_3        CAACCTTGTTTCTGAGCATGAACGCCCGCAAGCCAACATGTTAGTTGAAGCATCAGGGCG   260
B4387_1      CAACCTTGTTTCTGAGCATGAACGCCCGCAAGCCAACATGTTAGTTGAAGCATCAGGGCG   260
B4387_2      CAACCTTGTTTCTGAGCATGAACGCCCGCAAGCCAACATGTTAGTTGAAGCATCAGGGCG   260
A287_1       CAC---TCTTACCGAACTTGGCC----GTAACCTGCCA------------ACCAAAGCG   272
B73_1        CAC---TCTTACCGAACTTGGCC----GTAACCTGCCA------------ACCAAAGCG   272
B73_2        CAC---TCTTACCGAACTTGGCC----GTAACCTGCCA------------ACCAAAGCG   272
B4387_3      CAC---TCTTACCGAACTTGGCC----GTAACCTGCCA------------ACCAAAGCG   271
             **    * ** * ** * **  *    * ** *   **             * **  ***
```

```
B73_3        ATT-AGCAGCATGATATCAAAACGCTCTGAGCTGCTCGTTCGGCTATGGCGTAGGCCTAG   319
B4387_1      ATT-AGCAGCATGATATCAAAACGCTCTGAGCTGCTCGTTCGGCTATGGCGTAGGCCTAG   319
B4387_2      ATT-AGCAGCATGATATCAAAACGCTCTGAGCTGCTCGTTCGGCTATGGCGTAGGCCTAG   319
A287_1       AGAACAAAACATAACATCAAACGA--ATCGACCGATTGTTAGGTAATCGTCACCTCCACA   330
B73_1        AGAACAAAACATAACATCAAACGA--ATCGACCGATTGTTAGGTAATCGTCACCTCCACA   330
B73_2        AGAACAAAACATAACATCAAACGA--ATCGACCGATTGTTAGGTAATCGTCACCTCCACA   330
B4387_3      AGAACAAAACATAACATCAAACGA--ATCGACCGATTGTTAGGTAATCGTCACCTCCACA   329
             *      * *** * ******      *   * * * *** **  ** *      **

B73_3        TCCGTAGGCAGGACTTTTCAAGTCTCGGAAGGTTTCTTCAATCTGC--ATTCGCTTCGAA   377
B4387_1      TCCGTAGGCAGGACTTTTCAAGTCTCGGAAGGTTTCTTCAATCTGC--ATTCGCTTCGAA   377
B4387_2      TCCGTAGGCAGGACTTTTCAAGTCTCGGAAGGTTTCTTCAATCTGC--ATTCGCTTCGAA   377
A287_1       AAGAGCGACT-CGC-TGTATACCGTTGGCATGCTAGCTTTATCTGTTCGGGCAATACGAT   388
B73_1        AAGAGCGACT-CGC-TGTATACCGTTGGCATGCTAGCTTTATCTGTTCGGGCAATACGAT   388
B73_2        AAGAGCGACT-CGC-TGTATACCGTTGGCATGCTAGCTTTATCTGTTCGGGCAATACGAT   388
B4387_3      AAGAGCGACT-CGC-TGTATACCGTTGGCATGCTAGCTTTATCTGTTCGGGCAATACGAT   387
                   * *    * * *  *   * ** * * *   *  *****      *  * ***

B73_3        TAGATATTAACAAGTTGTTTGGGTGTTCGAATTTCAACAGGTAAGTTAGTTGCTAGAACC   437
B4387_1      TAGATATTAACAAGTTGTTTGGGTGTTCGAATTTCAACAGGTAAGTTAGTTGCTAGAATC   437
B4387_2      TAGATATTAACAAGTTGTTTGGGTGTTCGAATTTCAACAGGTAAGTTAGTTGCTAGAATC   437
A287_1       GCCCATTGTACTTGTTGAC---TGGTCTGATATCCGTGAGCAAAAACGGCTTATGGTATT   445
B73_1        GCCCATTGTACTTGTTGAC---TGGTCTGATATCCGTGAGCAAAAACGGCTTATGGTATT   445
B73_2        GCCCATTGTACTTGTTGAC---TGGTCTGATATCCGTGAGCAAAAACGGCTTATGGTATT   445
B4387_3      GCCCATTGTACTTGTTGAC---TGGTCTGATATTCGTGAGCAAAAACGACTTATGGTATT   444
                   *  **  ****       **  **  * *   **  **      *  * * *

B73_3        CATGGCTCCTTTGCCGACGCTGAGTAGATTTTAGGTGACGGGTGGTGACAATGAGTCCGT   497
B4387_1      CATGGCTCCTTTGCCGACGCTGAGTAGATTTTAGGTGACGGGTGGTGACAATGAGTCCGT   497
B4387_2      CATGGCTCCTTTGCCGACGCTGAGTAGATTTTAGGTGACGGGTGGTGACAATGAGTCCGT   497
A287_1       GCGAGCTTCAG---TCGCACTACACGGTCGTTCTGTTACTCTTTATGAGAAAGCGTTCCC   502
B73_1        GCGAGCTTCAG---TCGCACTACACGGTCGTTCTGTTACTCTTTATGAGAAAGCGTTCCC   502
B73_2        GCGAGCTTCAG---TCGCACTACACGGTCGTTCTGTTACTCTTTATGAGAAAGCGTTCCC   502
B4387_3      GCGAGCTTCAG---TCGCACTACACGGTCGTTCTGTTACTCTTTATGAGAAAGCGTTCCC   501
                 *** *        * **     *   **  ** **   *  *** ** * ** *

B73_3        G--TCGAGCGCTGATTTTT---------------------TCGGCCTTTAGAGCGAGATT   534
B4387_1      G--TCGAGCGCTGATTTTT---------------------TCGGCCTTTAGAGCGAGATT   534
B4387_2      G--TCGAGCGCTGATTTTT---------------------TCGGCCTTTAGAGCGAGATT   534
A287_1       GCTTTCAGAGCAATGTTCAAAGAAAGCTCATGACCAATTTCTAGCCGACCTTGCGAGCAT   562
B73_1        GCTTTCAGAGCAATGTTCAAAGAAAGCTCATGACCAATTTCTAGCCGACCTTGCGAGCAT   562
B73_2        GCTTTCAGAGCAATGTTCAAAGAAAGCTCATGACCAATTTCTAGCCGACCTTGCGAGCAT   562
B4387_3      GCTTTCAGAGCAATGTTCAAAGAAAGCTCATGACCAATTTCTAGCCGACCTTGCGAGCAT   561
             *  *  ** **    **                          ***      *****  *
```

```
B73_3       TATACAATAGA----------ATTTGGCATGAGATTGGATTGCTTTTAGTCAGCCTCTTA   584
B4387_1     TATACAATAGA----------ATTTGGCATGAGATTGGGTTGCTTTTAGTCAGCCTCTTA   584
B4387_2     TATACAATAGA----------ATTTGGCATGAGATTGGGTTGCTTTTAGTCAGCCTCTTA   584
A287_1      TCTACCGAGTAACACCACACCGCTCATTGTCAGTGATGCTGGCTTTAAAGT-----GCCA   617
B73_1       TCTACCGAGTAACACCACACCGCTCATTGTCAGTGATGCTGGCTTTAAAGT-----GCCA   617
B73_2       TCTACCGAGTAACACCACACCGCTCATTGTCAGTGATGCTGGCTTTAAAGT-----GCCA   617
B4387_3     TCTACCGAGTAACACCACACCGCTCATTGTCAGTGATGCTGGCTTTAAAGT-----GCCA   616
            * ***     *            *     * **    * * ***** *           *

B73_3       TAGCCTAAAGTCTTTGAGTGACTAGATGACATATCATGTAAGT---TGCTGATAGGTTTC   641
B4387_1     TAGCCTAAAGTCTTTGAGTGACTAGATGACATATCATGTAAGT---TGCTGATAGGTTTC   641
B4387_2     TAGCCTAAAGTCTTTGAGTGACTAGATGACATATCATGTAAGT---TGCTGATAGGTTTC   641
A287_1      TGGTATAAATCCGTTGAGAAGCTGGGTTGGTACTGGTTAAGTCGAGTAAGAGGAAAAGTA   677
B73_1       TGGTATAAATCCGTTGAGAAGCTGGGTTGGTACTGGTTAAGTCGAGTAAGAGGAAAAGTA   677
B73_2       TGGTATAAATCCGTTGAGAAGCTGGGTTGGTACTGGTTAAGTCGAGTAAGAGGAAAAGTA   677
B4387_3     TGGTATAAATCCGTTGAGAAGCTGGGTTGGTACTGGTTAAGTCGAGTAAGAGGAAAAGTA   676
            * *  ****  * *****   ** * *      *  *  *      *      *    *

B73_3       CAGTTTTCCGCTCCTAGGTCTGCATATTGTACTT-----TTCCTCTTACTCGACTTAACC   696
B4387_1     CAGTTTTCCGCTCCTAGGTCTGCATATTGTACTT-----TTCCTCTTACTCGACTTAACC   696
B4387_2     CAGTTTTCCGCTCCTAGGTCTGCATATTGTACTT-----TTCCTCTTACTCGACTTAACC   696
A287_1      CAATATGCAGACCTAGGAGCGGAAAACTGGAAACCTATCAGCAACTTACATGATATGTCA   737
B73_1       CAATATGCAGACCTAGGAGCGGAAAACTGGAAACCTATCAGCAACTTACATGATATGTCA   737
B73_2       CAATATGCAGACCTAGGAGCGGAAAACTGGAAACCTATCAGCAACTTACATGATATGTCA   737
B4387_3     CAATATGCAGACCTAGGAGCGGAAAACTGGAAACCTATCAGCAACTTACATGATATGTCA   736
            ** * * * *  *   *  * * * * ** *          *  *****  **  *  *

B73_3       AGTACCAACCCAGCTTCTCAACGGATTTATACCATGGCACTTTAAAGCCAGCATCACTGA   756
B4387_1     AGTACCAACCCAGCTTCTCAACGGATTTATACCATGGCACTTTAAAGCCAGCATCACTGA   756
B4387_2     AGTACCAACCCAGCTTCTCAACGGATTTATACCATGGCACTTTAAAGCCAGCATCACTGA   756
A287_1      --------TCTAGTCACTCAAAGACTTTAGGCTATAAGAGGC---------------TGA   774
B73_1       --------TCTAGTCACTCAAAGACTTTAGGCTATAAGAGGC---------------TGA   774
B73_2       --------TCTAGTCACTCAAAGACTTTAGGCTATAAGAGGC---------------TGA   774
B4387_3     --------TCTAGTCACTCAAAGACTTTAGGCTATAAGAGGC---------------TGA   773
                     * **   ***** *  ****  * **   *                  ***

B73_3       CAATGAGCGGTGTGGTGTTACTCGGTAGAATGCTCGCAAGGTCGGCTAGAAATTGGTCAT   816
B4387_1     CAATGAGCGGTGTGGTGTTACTCGGTAGAATGCTCGCAAGGTCGGCTAGAAATTGGTCAT   816
B4387_2     CAATGAGCGGTGTGGTGTTACTCGGTAGAATGCTCGCAAGGTCGGCTAGAAATTGGTCAT   816
A287_1      CTAAAAG------------------------CAACCCAATCTC---------ATGCCAAA   801
B73_1       CTAAAAG------------------------CAACCCAATCTC---------ATGCCAAA   801
B73_2       CTAAAAG------------------------CAACCCAATCTC---------ATGCCAAA   801
B4387_3     CTAAAAG------------------------CAATCCAATCTC---------ATGCCAAA   800
            * *  **                             ***  **          **   *
```

```
B73_3       GAGCTTTCTTTGAACATTGCTCTGAAAGCGGGAAC--------------------GCTTT   856
B4387_1     GAGCTTTCTTTGAACATTGCTCTGAAAGCGGGAAC--------------------GCTTT   856
B4387_2     GAGCTTTCTTTGAACATTGCTCTGAAAGCGGGAAC--------------------GCTTT   856
A287_1      TTCTATTGTATAAATCTCGCTCTAAAGGCCGAAAAAATCAGCGCTCGACACGGACTCATT   861
B73_1       TTCTATTGTATAAATCTCGCTCTAAAGGCCGAAAAAATCAGCGCTCGACACGGACTCATT   861
B73_2       TTCTATTGTATAAATCTCGCTCTAAAGGCCGAAAAAATCAGCGCTCGACACGGACTCATT   861
B4387_3     TTCTATTGTATAAATCTCGCTCTAAAGGCCGAAAAAATCAGCGCTCGACACGGACTCATT   860
                 ** * * **  * ***** ** ** * **                      * **

B73_3       CTCATAAAGAGTAACAGAACGACCGTGTAGT---GCGACTGAAGCTCGCAATACCATAAG   913
B4387_1     CTCATAAAGAGTAACAGAACGACCGTGTAGT---GCGACTGAAGCTCGCAATACCATAAG   913
B4387_2     CTCATAAAGAGTAACAGAACGACCGTGTAGT---GCGACTGAAGCTCGCAATACCATAAG   913
A287_1      GTCACCACCCGTCACCTAAAATCTACTCAGCGTCGGCAAAGGAGCCATGGATTCTAGCAA   921
B73_1       GTCACCACCCGTCACCTAAAATCTACTCAGCGTCGGCAAAGGAGCCATGGATTCTAGCAA   921
B73_2       GTCACCACCCGTCACCTAAAATCTACTCAGCGTCGGCAAAGGAGCCATGGATTCTAGCAA   921
B4387_3     GTCACCACCCGTCACCTAAAATCTACTCAGCGTCGGCAAAGGAGCCATGGGTTCTAGCAA   920
             ***  *   ** **  **   *     **    *  *  * ***      * * *  *

B73_3       TCGTTTTTGCTCACGAATATCAGACCAGTCAAC---AAGTACAATGGGCATCGTATTGCC   970
B4387_1     CCGTTTTTGCTCACGGATATCAGACCAGTCAAC---AAGTACAATGGGCATCGTATTGCC   970
B4387_2     CCGTTTTTGCTCACGGATATCAGACCAGTCAAC---AAGTACAATGGGCATCGTATTGCC   970
A287_1      CTAACTTACCTGTTGAAATTCGAACACCCAAACAACTTGTTAATATCTATTCGAAGC--G   979
B73_1       CTAACTTACCTGTTGAAATTCGAACACCCAAACAACTTGTTAATATCTATTCGAAGC--G   979
B73_2       CTAACTTACCTGTTGAAATTCGAACACCCAAACAACTTGTTAATATCTATTCGAAGC--G   979
B4387_3     CTAACTTACCTGTTGAAATTCGAACACCCAAACAACTTGTTAATATCTATTCGAAGC--G   978
                 **  **   * *  **  **     ***     **  *       *** *

B73_3       CGAACAGATAAAGCTAGCATGCCAACGGTATACAGCGAGTCGCT-CTTTGTG-GAGGTGA   1028
B4387_1     CGAACAGATAAAGCTAGCATGCCAACGGTATACAGCGAGTCGCT-CTTTGTG-GAGGTGA   1028
B4387_2     CGAACAGATAAAGCTAGCATGCCAACGGTATACAGCGAGTCGCT-CTTTGTG-GAGGTGA   1028
A287_1      AATGCAGATTGAAGAAACCTTCCGAGACTTGAAAAGTCCTGCCTACGGACTAGGCCTACG   1039
B73_1       AATGCAGATTGAAGAAACCTTCCGAGACTTGAAAAGTCCTGCCTACGGACTAGGCCTACG   1039
B73_2       AATGCAGATTGAAGAAACCTTCCGAGACTTGAAAAGTCCTGCCTACGGACTAGGCCTACG   1039
B4387_3     AATGCAGATTGAAGAAACCTTCCGAGACTTGAAAAGTCCTGCCTACGGACTAGGCCTACG   1038
                *****  *   * * * ** *   *  * *     *  ** *    *  *

B73_3       CGATTACCTAACAATCG-GTCGATTCGT-TTGATGTTATGTTTTGTTCTCGCTTTGG---   1083
B4387_1     CGATTACCTAACAATCG-GTCGATTCGT-TTGATGTTATGTTTTGTTCTCGCTTTGG---   1083
B4387_2     CGATTACCTAACAATCG-GTCGATTCGT-TTGATGTTATGTTTTGTTCTCGCTTTGG---   1083
A287_1      CCATAGCCGAACGAGCAGCTCAGAGCGTTTTGATATCATGC-TGCTAATCGCCCTGATGC   1098
B73_1       CCATAGCCGAACGAGCAGCTCAGAGCGTTTTGATATCATGC-TGCTAATCGCCCTGATGC   1098
B73_2       CCATAGCCGAACGAGCAGCTCAGAGCGTTTTGATATCATGC-TGCTAATCGCCCTGATGC   1098
B4387_3     CCATAGCCGAACGAGCAGCTCAGAGCGTTTTGATATCATGC-TGCTAATCGCCCTGATGC   1097
            * **  ** *** * *   **    *** ***** * ***  *  *  ****  **
```

```
B73_3           ----------TTGGCAGGTTACGGCCAAGTTCGGTAAGAGTGAGAGTTTTACAGTCAAGT   1133
B4387_1         ----------TTGGCAGGTTACGGCCAAGTTCGGTAAGAGTGAGAGTTTTACAGTCAAGT   1133
B4387_2         ----------TTGGCAGGTTACGGCCAAGTTCGGTAAGAGTGAGAGTTTTACAGTCAAGT   1133
A287_1          TTCAACTAACATGTTGGCTTGCGGGCGTTCATGCTCAGAAACAAGGTTG---GGACAAGC   1155
B73_1           TTCAACTAACATGTTGGCTTGCGGGCGTTCATGCTCAGAAACAAGGTTG---GGACAAGC   1155
B73_2           TTCAACTAACATGTTGGCTTGCGGGCGTTCATGCTCAGAAACAAGGTTG---GGACAAGC   1155
B4387_3         TTCAACTAACATGTTGGCTTGCGGGCGTTCATGCTCAGAAACAAGGTTG---GGACAAGC   1154
                           **   * ** *** *      * * ***   *  ***     * ****

B73_3           AATGCGTGGCAAGCCAACGTTAAGCTGTTGAGTCGTTTTAAGTGTAATTCG-----GGGC   1188
B4387_1         AAGGCGTGGCAAGCCAACGTTAAGCTGTTGAGTCGTTTTAAGTGTAATTCG-----GGGC   1188
B4387_2         AAGGCGTGGCAAGCCAACGTTAAGCTGTTGAGTCGTTTTAAGTGTAATTCG-----GGGC   1188
A287_1          ACTTCCAGGCTAACACA--GTCAGAAATCGAAACGTACTCTCAACAGTTCGCTTAGGCAT   1213
B73_1           ACTTCCAGGCTAACACA--GTCAGAAATCGAAACGTACTCTCAACAGTTCGCTTAGGCAT   1213
B73_2           ACTTCCAGGCTAACACA--GTCAGAAATCGAAACGTACTCTCAACAGTTCGCTTAGGCAT   1213
B4387_3         ACTTCCAGGCTAACACA--GTCAGAAATCGAAACGTACTCTCAACAGTTCGCTTAGGCAT   1212
                *   *  *** * *  *   * **   * **  ***  *      * ****     *

B73_3           AGAATTGGTAAAGAGAGTCG--TGTAAAATATCGAGTTCGCACATCTTGTTGTCTGATTA   1246
B4387_1         AGAATTGGTAAAGAGAGTCG--TGTAAAATATCGAGTTCGCACATTTTGTTGTCTGATTA   1246
B4387_2         AGAATTGGTAAAGAGAGTCG--TGTAAAATATCGAGTTCGCACATTTTGTTGTCTGATTA   1246
A287_1          GGAAGTTTTGCGGCATTCTGGCTACACAATAACAAGGGAAGACTCACTCGTGGCTGCAAC   1273
B73_1           GGAAGTTTTGCGGCATTCTGGCTACACAATAACAAGGGAAGACTCACTCGTGGCTGCAAC   1273
B73_2           GGAAGTTTTGCGGCATTCTGGCTACACAATAACAAGGGAAGACTCACTCGTGGCTGCAAC   1273
B4387_3         GGAAGTTTTGCGGCATTCTGGCTACACAATAACAAGGGAAGACTTACTCGTGGCTGCAAC   1272
                 *** *  *   *      *  *  * **** * **     **    *  ** ***

B73_3           TTGATTTTTCGCGAAACCATTTGATCATATGACAAGATGTGTATCCACCTTAACTTAATG   1306
B4387_1         TTGATTTTTGGCGAAACCATTTGATCATATGACAAGATGTGTATCTACCTTAACTTAATG   1306
B4387_2         TTGATTTTTGGCGAAACCATTTGATCATATGACAAGATGTGTATCTACCTTAACTTAATG   1306
A287_1          CC--TGCTTACTCAA----------------------AATCTATTCACA---CATGGTTA   1306
B73_1           CC--TGCTTACTCAA----------------------AATCTATTCACA---CATGGTTA   1306
B73_2           CC--TGCTTACTCAA----------------------AATCTATTCACA---CATGGTTA   1306
B4387_3         CC--TACTAGCTCAA----------------------AATTTATTCACA---CATGGTTA   1305
                    *  *     **                        * ***  **      *   *

B73_3           ATTTTTACCAAAATCATTAGGGGATTCATCAG     1338
B4387_1         ATTTTGATAAAAATCATTAGGGGATTCATCAG     1338
B4387_2         ATTTTGATAAAAATCATTAGGGGATTCATCAG     1338
A287_1          CGTTTTGGGGAAATTATGAGGGGATCTCTCAG     1338
B73_1           CGTTTTGGGGAAATTATGAGGGGATCTCTCAG     1338
B73_2           CGTTTTGGGGAAATTATGAGGGGATCTCTCAG     1338
B4387_3         CGCTTTGGGGAAATTATGAGGGGATCTCTCAG     1337
                   **     **** ** *******   ****
```

Table S2. Bacterial strains

Strain	Relevant phenotype or genotype*	Source/Reference
NEB5a		New England Biolab
MIT A2	*pks*$^{+}$	Mouse isolate, from the James Fox lab at MIT
MIT A21	*pks*$^{+}$	Mouse isolate, from the James Fox lab at MIT
MIT guinea pig isolate	*pks*$^{+}$	Guinea pig isolate, from the James Fox lab at MIT
NC101	*pks*$^{+}$	JANELLE C. ARTHUR, et al., 2012
NC101^{CmR}	*pks*$^{+}$ with a Cm resistance gene	This study
NC101 Δ*pks*	*pks* knockout	JANELLE C. ARTHUR, et al., 2012
DH10B pBAC	pBAC vector containing the *pks* island	Jean-Philippe Nougayrède, et al., 2021
DH10B pBAC-*pks*	Empty pBAC vector	Jean-Philippe Nougayrède, et al., 2021
EC100Dpir^{+}	F– mcrA Δ(mrr-hsdRMS-mcrBC) φ80dlacZΔM15 ΔlacX74 recA1 endA1 araD139 Δ(ara, leu)7697 galU galK λ– rpsL nupG pir+ (DHFR)	#ECP09500 (Lucigen)

MG1655^{NxR}	K-12 F– λ– ilvG– rfb-50 rph-1 Nxr	(Carraro et al., 2014)
E. coli Nissle 1917 strepR	*pks*$^{+}$ and Streptomycin resistance	Jean-Philippe Nougayrède, et al., 2021
eB-TP114	Evolved by accelerated laboratory evolution for 5 cycles in broth	This study
KN01	Smr, Spr Nissle 1917	Neil, et al., 2020

Table S3: Replicative and conjugative plasmids

Plasmid	Relevant phenotype or genotype*	Source/Reference
pBXB1	oriVpMB1, bxb1 integrase, bla (Apr)	Genbank: MK756311 (Neil et al., 2019)
pE-FLP	oriVpSC101ts, flp, Apr	Addgene #45978
pGRG25: CmR	Tn7 machinery to insert the aad7 and cat genes in the glmS terminator region	This study
pKill1	oriVpSC101ts, attPbxb1, FRT, 1 gRNA vs cat, aph-IIIa (Kmr), cas9	Genbank: MK756312 (Neil et al., 2019)
pNative-Cas9	Adapted from pKill1	This study
pNative-dCas9	oriVpSC101ts, attPbxb1, FRT, 1 gRNA vs cat, aph-IIIa (Kmr), dcas9	This study
pBAD-dCas9	oriVpSC101ts, attPbxb1, FRT, 1 gRNA vs cat, aph-IIIa (Kmr), dcas9, pBAD proter	This study
eB-TP114::tetB	eB-TP114Δaph-III::tetB	(Neil et al., 2021)
eB-TP114::pNative-Cas9	eB-TP114::tetB with inserted wild-type Cas9 with the original native promoter after bxb1- and flp-mediated deletion of tetB and oriVpSC101ts	This study
eB-TP114::pNative-dCas9	eB-TP114::tetB with inserted catalytically inactive Cas9 (dCas9) and the original native promoter after bxb1- and flp-mediated deletion of tetB and oriVpSC101ts	This study
eB-TP114::pBAD-dCas9	eB-TP114::tetB with dCas9 and the arabinose inducible promoter, pBAD, after bxb1- and flp-mediated deletion of tetB and oriVpSC101ts	This study

Table S4: Cloning, qPCR and sequencing primers

Capitalized letters in the sgRNA primers are the 20-nt spacer.

Name	Sequence	Descriptions
clbP_F	tatcatctcctgtgctgtatgctg	qPCR
clbP_R	cctgcatccgttggtgaatt	qPCR
Universal 16S_F	ggtgaatacgttcccgg	qPCR
Universal 16S_R	tacggctaccttgttacgactt	qPCR
irp1_F1	cactttctccgctaaggcca	qPCR
irp1_R1	gcatcattcacgatccgcac	qPCR
irp1_F2	agcacgttgtccatccagta	qPCR
irp1_R2	cggcgaaccctgctatgtat	qPCR
irp1_F3	aacagggtttcctcaccgtc	qPCR
irp1_R3	ttgccagggctaaccttcag	qPCR
clbA_287_fwd	GGCTACTGTATCTATAGTATgttttagagctagaaatagcaagt	sgRNA primers
clbA_287_rev	ATACTATAGATACAGTAGCCgaatctattatacagaaaaattttcctga	sgRNA primers
clbB_73_fwd	AATAAAAACCCCTGTTGACTgttttagagctagaaatagcaagt	sgRNA primers
clbB_73_rev	AGTCAACAGGGGTTTTTATTgaatctattatacagaaaaattttcctga	sgRNA primers
clbB_1667_fwd	TGGCAACCACGGTGTTTACCgttttagagctagaaatagcaagt	sgRNA primers
clbB_1667_rev	GGTAAACACCGTGGTTGCCAgaatctattatacagaaaaattttcctga	sgRNA primers
clbB_5400_fwd	TGTGCAAAATCGTGCAGCATgttttagagctagaaatagcaagt	sgRNA primers
clbB_5400_rev	ATGCTGCACGATTTTGCACAgaatctattatacagaaaaattttcctga	sgRNA primers
clbB_3673_fwd	GGAATGGATATTCAGGATACgttttagagctagaaatagcaagt	sgRNA primers
clbB_3673_rev	GTATCCTGAATATCCATTCCgaatctattatacagaaaaattttcctga	sgRNA primers
clbB_4387_fwd	GAACGCGATAGATCTATAGCgttttagagctagaaatagcaagt	sgRNA primers
clbB_4387_rev	GCTATAGATCTATCGCGTTCgaatctattatacagaaaaattttcctga	sgRNA primers
clbB_5610_fwd	AAGGTAGAGAGCGTATTACCgttttagagctagaaatagcaagt	sgRNA primers
clbB_5610_rev	GGTAATACGCTCTCTACCTTgaatctattatacagaaaaattttcctga	sgRNA primers
clbB_4129_fwd	GATACTGTCGCTATCAAAACgttttagagctagaaatagcaagt	sgRNA primers
clbB_4129_rev	GTTTTGATAGCGACAGTATCgaatctattatacagaaaaattttcctga	sgRNA primers

clbC_516_fwd	CTGGTGTAGAGACTATACACgttttagagctagaaatagcaagt	sgRNA primers
clbC_516_rev	GTGTATAGTCTCTACACCAGgaatctattatacagaaaaattttcc tga	sgRNA primers
clbC_2085_fwd	TCAACGTCTTCTCTTATTTCgttttagagctagaaatagcaagt	sgRNA primers
clbC_2085_rev	GAAATAAGAGAAGACGTTGAgaatctattatacagaaaaattttc ctga	sgRNA primers
clbC_2313_fwd	ACGAAAGGTACGCTTAACACgttttagagctagaaatagcaagt	sgRNA primers
clbC_2313_rev	GTGTTAAGCGTACCTTTCGTgaatctattatacagaaaaattttcct ga	sgRNA primers
clbC_1481_fwd	CGTTTTCCACTTGTATCACTgttttagagctagaaatagcaagt	sgRNA primers
clbC_1481_rev	AGTGATACAAGTGGAAAACGgaatctattatacagaaaaattttc ctga	sgRNA primers
clbH_1167_fwd	CCAGTTTTATACAGCCGCTCgttttagagctagaaatagcaagt	sgRNA primers
clbH_1167_rev	GAGCGGCTGTATAAAACTGGgaatctattatacagaaaaattttc ctga	sgRNA primers
clbH_1297_fwd	CCCCGGGAAACGCATCGCATgttttagagctagaaatagcaagt	sgRNA primers
clbH_1297_rev	ATGCGATGCGTTTCCCGGGGgaatctattatacagaaaaattttcc tga	sgRNA primers
clbI_331_fwd	ACCCACATCCCCCGGATAATgttttagagctagaaatagcaagt	sgRNA primers
clbI_331_rev	ATTATCCGGGGGATGTGGGTgaatctattatacagaaaaattttc ctga	sgRNA primers
clbI_170_fwd	CTTTGGCCTTGACGTAATCTgttttagagctagaaatagcaagt	sgRNA primers
clbI_170_rev	AGATTACGTCAAGGCCAAAGgaatctattatacagaaaaattttc ctga	sgRNA primers
clbJ_5578_fwd	GCCCCGACCGTAGGGAATACgttttagagctagaaatagcaagt	sgRNA primers
clbJ_5578_rev	GTATTCCCTACGGTCGGGGCgaatctattatacagaaaaattttcc tga	sgRNA primers
clbJ_3688_fwd	CGTGCGCATACTCAGAGCATgttttagagctagaaatagcaagt	sgRNA primers
clbJ_3688_rev	ATGCTCTGAGTATGCGCACGgaatctattatacagaaaaattttcc tga	sgRNA primers
clbL_412_fwd	TAATAGCTCGTTATCCCCTCgttttagagctagaaatagcaagt	sgRNA primers
clbL_412_rev	GAGGGGATAACGAGCTATTAgaatctattatacagaaaaattttc ctga	sgRNA primers
clbL_654_fwd	CTGTTGATCTATCTCATGGTgttttagagctagaaatagcaagt	sgRNA primers
clbL_654_rev	ACCATGAGATAGATCAACAGgaatctattatacagaaaaattttc ctga	sgRNA primers
clbL_993_fwd	CACCGCCACCGCCGCTCCTCgttttagagctagaaatagcaagt	sgRNA primers
clbL_993_rev	GAGGAGCGGCGGTGGCGGTGgaatctattatacagaaaaattttc ctga	sgRNA primers
clbN_759_fwd	GCGCCAGCTTTCGTGTGTGGgttttagagctagaaatagcaagt	sgRNA primers
clbN_759_rev	CCACACACGAAAGCTGGCGCgaatctattatacagaaaaattttc ctga	sgRNA primers
irp1_4019_fwd	AACCTTCCCTAGCCACTCCGgttttagagctagaaatagcaag t	sgRNA primers
irp1_4019_rev	CGGAGTGGCTAGGGAAGGTTgaatctattatacagaaaaatttt cctga	sgRNA primers

irp1_4129_fwd	GGCGCAGGAAGAGGCTCGGCgttttagagctagaaatagcaagt	sgRNA primers
irp1_4129_rev	GCCGAGCCTCTTCCTGCGCCgaatctattatacagaaaaattttcctga	sgRNA primers
irp1_397_fwd	GTCTTATGGGGGACGGCGCCgttttagagctagaaatagcaagt	sgRNA primers
irp1_397_rev	GGCGCCGTCCCCCATAAGACgaatctattatacagaaaaattttcctga	sgRNA primers
irp1_1595_fwd	CCCCGATTTCTCACCGGCCCgttttagagctagaaatagcaagt	sgRNA primers
irp1_1595_rev	GGGCCGGTGAGAAATCGGGGgaatctattatacagaaaaattttcctga	sgRNA primers
irp1_ko_fwd	aacgtgttctccggttgcgttgaggttgcgcaaagccagcgggtaatgtgatgggaattagccatggtcc	irp1 knockout
irp1_ko_rev	aacgcatggcgttccattgactttatgaaccttaggaaatgggaccgattgtgtaggctggagctgcttc	iIrp1 knockout
BACT1369F	cggtgaatacgttcycgg	qPCR
PROK1541R	aaggaggtgatccrgccgca	qPCR
clbB_F1	acacccgctgcgttagagttgc	qPCR
clbB_R1	ccgatggtcaaaggctgtgcgt	qPCR
clbC_F1	gctggaaatatcgccggtcggg	qPCR
clbC_R1	ttgcgcggattggggttttcca	qPCR
1492R	tacggytaccttgttacgactt	16S whole sequence
27F	agagtttgatcmtggctcag	16S whole sequence
aph-III upstream TP114	ctatgccacgctacgccgctac	sequencing
aph-III downstream TP114	ctggtcacggcggcgaatatcc	sequencing
ClbA upstream	tcacgctatacacattgctaacag	*pks*$^{+}$ detection
ClbA downstream	tgtgaatggcacgattatgcggg	*pks*$^{+}$ detection
pkill1	gctcaacagtcacacatagacagcc	sequencing

Cm spacer in original pKill1	taacacgccacatcttgcga	sequencing
KO of clbP verification_dwn upstream	cgaatacggagcgacctcgatgc	*pks*$^{+}$ detection
KO of clbP verification_dwn	gtcagcgacggcatccaccatc	*pks*$^{+}$ detection
sgclbB_4387	gctatagatctatcgcgttc	sgRNA detection
sgclbC_2313	gtgttaagcgtacctttcgt	sgRNA detection
PstI_backbone	ggtgccctgaatgaactgca	Mutation of Cas9 to dCas9
D10A	tgtgccgatagctaagcctat	Mutation of Cas9 to dCas9
D10A	ataggcttagctatcggcaca	Mutation of Cas9 to dCas9
H840A	tggaacaatggcatcgacatc	Mutation of Cas9 to dCas9
H840A	gatgtcgatgccattgttcca	Mutation of Cas9 to dCas9
BamHI_backbone	actatcaaaaccaccatattttttg	Mutation of Cas9 to dCas9
pBAD_Promoter_fwd	accccagcttcaaaagcgctatcgatgcataatgtgcc	Construction of pBAD_dCas9
pBAD_Promoter_rev	attgagtatttcttatccatggtatatctccttattaaagttaaac	Construction of pBAD_dCas9
pKILL1-cas9_Backbone_fwd	atggataagaaatactcaataggcttag	Construction of pBAD_dCas9
pKILL1-cas9_Backbone_rev	agcgcttttgaagctggg	Construction of pBAD_dCas9

Table S5. Promoter and coding DNA sequences

Name	Length	sequences
pNative promoter	240 bp	tttatcagccataaaacaatacttaatactatagaatgataacaaaataaactactttttaaaagaattttgtgttata<br>atctatttattattaagtattgggtaatatttttttgaagagatattttgaaaaagaaaaattaaagcatattaaactaat<br>ttcggaggtcattaaaactattattgaaatcatcaaactcattatggatttaatttaaactttttattttaggaggcaa<br>aa
pBAD promoter	1346 bp	atcgatgcataatgtgcctgtcaaatggacgaagcagggattctgcaaaccctatgctactccgtcaagccgt<br>caattgtctgattcgttaccaattatgacaacttgacggctacatcattcactttttcttcacaaccggcacggaac<br>tcgctcgggctggccccggtgcattttttaaatacccgcgagaaatagagttgatcgtcaaaaccaacattgcg<br>accgacggtggcgataggcatccgggtggtgctcaaaagcagcttcgcctggctgatacgttggtcctcgcg<br>ccagcttaagacgctaatccctaactgctggcggaaaagatgtgacagacgcgacggcgacaagcaaacat<br>gctgtgcgacgctggcgatatcaaaattgctgtctgccaggtgatcgctgatgtactgacaagcctcgcgtac<br>ccgattatccatcggtggatggagcgactcgttaatcgcttccatgcgccgcagtaacaattgctcaagcagat<br>ttatcgccagcagctccgaatagcgcccttccccttgcccggcgttaatgatttgcccaaacaggtcgctgaaa<br>tgcggctggtgcgcttcatccgggcgaaagaaccccgtattggcaaatattgacggccagttaagccattcat<br>gccagtaggcgcgcggacgaaagtaaacccactggtgataccattcgcgagcctccggatgacgaccgta<br>gtgatgaatctctcctggcgggaacagcaaaatatcacccggtcggcaaacaaattctcgtccctgatttttca<br>ccaccccctgaccgcgaatggtgagattgagaatataacctttcattcccagcggtcggtcgataaaaaaatc<br>gagataaccgttggcctcaatcggcgttaaacccgccaccagatgggcattaaacgagtatcccggcagca<br>ggggatcattttgcgcttcagccatacttttcatactcccgccattcagagaagaaaccaattgtccatattgcat<br>cagacattgccgtcactgcgtcttttactggctcttctcgctaaccaaaccggtaaccccgcttattaaaagcatt<br>ctgtaacaaagcgggaccaaagccatgacaaaaacgcgtaacaaaagtgtctataatcacggcagaaaagt<br>ccacattgattatttgcacggcgtcacactttgctatgccatagcatttttatccataagattagcggatcctacct<br>gacgctttttatcgcaactctctactgtttctccatacccgtttttttgggctagccctgtagaaataattgtttaactt<br>taataaggagatatacc
Cas9	4107 bp	atggataagaaatactcaataggcttagatatcggcacaaatagcgtcggatgggcggtgatcactgatgaat<br>ataaggttccgtctaaaaagttcaaggttctgggaaatacagaccgccacagtatcaaaaaaaatcttataggg<br>gctcttttatttgacagtggagagacagcggaagcgactcgtctcaaacggacagctcgtagaaggtatacac<br>gtcggaagaatcgtatttgttatctacaggagatttttcaaatgagatggcgaaagtagatgatagtttctttcat<br>cgacttgaagagtcttttttggtggaagaagacaagaagcatgaacgtcatcctatttttggaaatatagtagat<br>gaagttgcttatcatgagaaatatccaactatctatcatctgcgaaaaaaattggtagattctactgataaagcgg<br>atttgcgcttaatctatttggccttagcgcatatgattaagtttcgtggtcatttttttgattgagggagatttaaatcct<br>gataatagtgatgtggacaaactatttatccagttggtacaaacctacaatcaattatttgaagaaaaccctatta<br>acgcaagtggagtagatgctaaagcgattctttctgcacgattgagtaaatcaagacgattagaaaatctcattg<br>ctcagctccccggtgagaagaaaaatggcttatttgggaatctcattgctttgtcattgggtttgacccctaatttt<br>aaatcaaattttgatttggcagaagatgctaaattacagctttcaaaagatacttacgatgatgatttagataattta<br>ttggcgcaaattggagatcaatatgctgatttgtttttggcagctaagaatttatcagatgctattttactttcagata<br>tcctaagagtaaatactgaaataactaaggctcccctatcagcttcaatgattaaacgctacgatgaacatcatc<br>aagacttgactcttttaaaagctttagttcgacaacaacttccagaaaagtataaagaaatcttttttgatcaatca<br>aaaaacggatatgcaggttatattgatgggggagctagccaagaagaattttataaatttatcaaaccaattta<br>gaaaaaatggatggtactgaggaattattggtgaaactaaatcgtgaagatttgctgcgcaagcaacggacct<br>ttgacaacggctctattccccatcaaattcacttgggtgagctgcatgctattttgagaagacaagaagactttta<br>tccatttttaaaagacaatcgtgagaagattgaaaaaatcttgacttttcgaattccttattatgttggtccattggc<br>gcgtggcaatagtcgttttgcatggatgactcggaagtctgaagaaacaattaccccatggaattttgaagaag<br>ttgtcgataaaggtgcttcagctcaatcatttattgaacgcatgacaaactttgataaaaatcttccaaatgaaaa<br>agtactaccaaaacatagtttgctttatgagtattttacggtttataacgaattgacaaaggtcaaatatgttactga<br>aggaatgcgaaaaccagcatttctttcaggtgaacagaagaaagccattgttgatttactcttcaaaacaaatcg<br>aaaagtaaccgttaagcaattaaaagaagattatttcaaaaaaatagaatgttttgatagtgttgaaatttcagga

		gttgaagatagatttaatgcttcattaggtacctaccatgatttgctaaaaattattaaagataaagatttttggata<br>atgaagaaaatgaagatatcttagaggatattgttttaacattgaccttatttgaagatagggagatgattgagga<br>aagacttaaaacatatgctcacctctttgatgataaggtgatgaaacagcttaaacgtcgccgttatactggttg<br>gggacgtttgtctcgaaaattgattaatggtattagggataagcaatctggcaaaacaatattagatttttgaaat<br>cagatggttttgccaatcgcaattttatgcagctgatccatgatgatagtttgacatttaaagaagacattcaaaa<br>agcacaagtgtctggacaaggcgatagtttacatgaacatattgcaaatttagctggtagccctgctattaaaaa<br>aggtattttacagactgtaaaagttgttgatgaattggtcaaagtaatggggcggcataagccagaaaatatcgt<br>tattgaaatggcacgtgaaaatcagacaactcaaaagggccagaaaaattcgcgagagcgtatgaaacgaat<br>cgaagaaggtatcaaagaattaggaagtcagattcttaaagagcatcctgttgaaaatactcaattgcaaaatg<br>aaaagctctatctctattatctccaaaatggaagagacatgtatgtggaccaagaattagatattaatcgtttaagt<br>gattatgatgtcgatcacattgttccacaaagtttccttaaagacgattcaatagacaataaggtcttaacgcgttc<br>tgataaaaatcgtggtaaatcggataacgttccaagtgaagaagtagtcaaaaagatgaaaaactattggaga<br>caacttctaaacgccaagttaatcactcaacgtaagtttgataatttaacgaaagctgaacgtggaggtttgagt<br>gaacttgataaagctggttttatcaaacgccaattggttgaaactcgccaaatcactaagcatgtggcacaaatt<br>ttggatagtcgcatgaatactaaatacgatgaaaatgataaacttattcgagaggttaaagtgattaccttaaaat<br>ctaaattagtttctgacttccgaaaagatttccaattctataaagtacgtgagattaacaattaccatcatgcccat<br>gatgcgtatctaaatgccgtcgttggaactgctttgattaagaaatatccaaaacttgaatcggagtttgtctatg<br>gtgattataaagtttatgatgttcgtaaaatgattgctaagtctgagcaagaaataggcaaagcaaccgcaaaat<br>atttcttttactctaatatcatgaacttcttcaaaacagaaattacacttgcaaatggagagattcgcaaacgccct<br>ctaatcgaaactaatggggaaactggagaaattgtctgggataaagggcgagattttgccacagtgcgcaaa<br>gtattgtccatgccccaagtcaatattgtcaagaaaacagaagtacagacaggcggattctccaaggagtcaa<br>ttttaccaaaaagaaattcggacaagcttattgctcgtaaaaaagactgggatccaaaaaaatatggtggttttg<br>atagtccaacggtagcttattcagtcctagtggttgctaaggtggaaaaagggaaatcgaagaagttaaaatcc<br>gttaaagagttactagggatcacaattatggaaagaagttcctttgaaaaaaatccgattgactttttagaagcta<br>aaggatataaggaagttaaaaaagacttaatcattaaactacctaaatatagtctttttgagttagaaaacggtcg<br>taaacggatgctggctagtgccggagaattacaaaaaggaaatgagctggctctgccaagcaaatatgtgaa<br>tttttatatttagctagtcattatgaaaagttgaagggtagtccagaagataacgaacaaaaacaattgtttgtgg<br>agcagcataagcattatttagatgagattattgagcaaatcagtgaattttctaagcgtgttattttagcagatgcc<br>aatttagataaagttcttagtgcatataacaaacatagagacaaaccaatacgtgaacaagcagaaaatattatt<br>catttatttacgttgacgaatcttggagctcccgctgcttttaaatattttgatacaacaattgatcgtaaacgatata<br>cgtctacaaaagaagttttagatgccactcttatccatcaatccatcactggtctttatgaaacacgcattgatttg<br>agtcagctaggaggtgactga
dCas9	4107 bp	atggataagaaatactcaataggcttaGCTatcggcacaaatagcgtcggatgggcggtgatcactgatga<br>atataaggttccgtctaaaaagttcaaggttctgggaaatacagaccgccacagtatcaaaaaaaatcttatag<br>gggctcttttatttgacagtggagagacagcggaagcgactcgtctcaaacggacagctcgtagaaggtata<br>cacgtcggaagaatcgtatttgttatctacaggagatttttcaaatgagatggcgaaagtagatgatagtttcttt<br>catcgacttgaagagtctttttggtggaagaagacaagaagcatgaacgtcatcctatttttggaaatatagtag<br>atgaagttgcttatcatgagaaatatccaactatctatcatctgcgaaaaaaattggtagattctactgataaagc<br>ggatttgcgcttaatctatttggccttagcgcatatgattaagtttcgtggtcatttttgattgagggagatttaaat<br>cctgataatagtgatgtggacaaactatttatccagttggtacaaacctacaatcaattatttgaagaaaaccta<br>ttaacgcaagtggagtagatgctaaagcgattctttctgcacgattgagtaaatcaagacgattagaaaatctca<br>ttgctcagctccccggtgagaagaaaaatggcttatttgggaatctcattgctttgtcattgggtttgacccctaat<br>tttaaatcaaattttgatttggcagaagatgctaaattacagctttcaaaagatacttacgatgatgatttagataatt<br>tattggcgcaaattggagatcaatatgctgatttgtttttggcagctaagaatttatcagatgctattttactttcaga<br>tatcctaagagtaaatactgaaataactaaggctcccctatcagcttcaatgattaaacgctacgatgaacatca<br>tcaagacttgactcttttaaaagctttagttcgacaacaacttccagaaaagtataaagaaatcttttttgatcaatc<br>aaaaaacggatatgcaggttatattgatgggggagctagccaagaagaattttataaatttatcaaaccaatttta<br>gaaaaaatggatggtactgaggaattattggtgaaactaaatcgtgaagatttgctgcgcaagcaacggacct<br>ttgacaacggctctattccccatcaaattcacttgggtgagctgcatgctattttgagaagacaagaagactttta<br>tccatttttaaaagacaatcgtgagaagattgaaaaaatcttgacttttcgaattccttattatgttggtccattggc<br>gcgtggcaatagtcgttttgcatggatgactcggaagtctgaagaaacaattaccccatggaattttgaagaag

		ttgtcgataaaggtgcttcagctcaatcatttattgaacgcatgacaaactttgataaaaatcttccaaatgaaaa<br>agtactaccaaaacatagtttgctttatgagtattttacggtttataacgaattgacaaaggtcaaatatgttactga<br>aggaatgcgaaaaccagcatttctttcaggtgaacagaagaaagccattgttgatttactcttcaaaacaaatcg<br>aaaagtaaccgttaagcaattaaaagaagattatttcaaaaaaatagaatgttttgatagtgttgaaatttcagga<br>gttgaagatagatttaatgcttcattaggtacctaccatgatttgctaaaaattattaaagataaagatttttggata<br>atgaagaaaatgaagatatcttagaggatattgttttaacattgaccttatttgaagatagggagatgattgagga<br>aagacttaaaacatatgctcacctctttgatgataaggtgatgaaacagcttaaacgtcgccgttatactggttg<br>gggacgtttgtctcgaaaattgattaatggtattagggataagcaatctggcaaaacaatattagatttttgaaat<br>cagatggttttgccaatcgcaattttatgcagctgatccatgatgatagtttgacatttaaagaagacattcaaaa<br>agcacaagtgtctggacaaggcgatagtttacatgaacatattgcaaatttagctggtagccctgctattaaaaa<br>aggtattttacagactgtaaaagttgttgatgaattggtcaaagtaatggggcggcataagccagaaaatatcgt<br>tattgaaatggcacgtgaaaatcagacaactcaaaagggccagaaaaattcgcgagagcgtatgaaacgaat<br>cgaagaaggtatcaaagaattaggaagtcagattcttaaagagcatcctgttgaaaatactcaattgcaaaatg<br>aaaagctctatctctattatctccaaaatggaagagacatgtatgtggaccaagaattagatattaatcgtttaagt<br>gattatgatgtcgatGCCattgttccacaaagtttccttaaagacgattcaatagacaataaggtcttaacgcgt<br>tctgataaaaatcgtggtaaatcggataacgttccaagtgaagaagtagtcaaaaagatgaaaaactattgga<br>gacaacttctaaacgccaagttaatcactcaacgtaagtttgataatttaacgaaagctgaacgtggaggtttga<br>gtgaacttgataaagctggttttatcaaacgccaattggttgaaactcgccaaatcactaagcatgtggcacaa<br>attttggatagtcgcatgaatactaaatacgatgaaaatgataaacttattcgagaggttaaagtgattaccttaa<br>aatctaaattagtttctgacttccgaaaagatttccaattctataaagtacgtgagattaacaattaccatcatgcc<br>catgatgcgtatctaaatgccgtcgttggaactgctttgattaagaaatatccaaaacttgaatcggagtttgtcta<br>tggtgattataaagtttatgatgttcgtaaaatgattgctaagtctgagcaagaaataggcaaagcaaccgcaaa<br>atatttcttttactctaatatcatgaacttcttcaaaacagaaattacacttgcaaatggagagattcgcaaacgcc<br>ctctaatcgaaactaatggggaaactggagaaattgtctgggataaagggcgagattttgccacagtgcgcaa<br>agtattgtccatgccccaagtcaatattgtcaagaaaacagaagtacagacaggcggattctccaaggagtca<br>attttaccaaaaagaaattcggacaagcttattgctcgtaaaaaagactgggatccaaaaaaatatggtggtttt<br>gatagtccaacggtagcttattcagtcctagtggttgctaaggtggaaaaagggaaatcgaagaagttaaaat<br>ccgttaaagagttactagggatcacaattatggaaagaagttcctttgaaaaaaatccgattgactttttagaagc<br>taaaggatataaggaagttaaaaaagacttaatcattaaactacctaaatatagtctttttgagttagaaaacggt<br>cgtaaacggatgctggctagtgccggagaattacaaaaaggaaatgagctggctctgccaagcaaatatgtg<br>aatttttatatttagctagtcattatgaaaagttgaagggtagtccagaagataacgaacaaaaacaattgtttgt<br>ggagcagcataagcattatttagatgagattattgagcaaatcagtgaattttctaagcgtgttattttagcagatg<br>ccaatttagataaagttcttagtgcatataacaaacatagagacaaaccaatacgtgaacaagcagaaaatatt<br>attcatttatttacgttgacgaatcttggagctcccgctgcttttaaatattttgatacaacaattgatcgtaaacgat<br>atacgtctacaaaagaagttttagatgccactcttatccatcaatccatcactggtctttatgaaacacgcattgat<br>ttgagtcagctaggaggtgactga
